# The Role of Neuritin 1 in Synaptic Plasticity and Sensory Nerve Function: Integrator of Neurotrophic, Metabolic and Injury Signals

**DOI:** 10.1111/ejn.70493

**Published:** 2026-04-12

**Authors:** Jyoti Agrawal, Mar Vives Escola, Simon W. Jones, Victoria Chapman, Federico Dajas‐Bailador

**Affiliations:** ^1^ School of Life Sciences University of Nottingham Nottingham UK; ^2^ Arthritis UK Pain Centre University of Nottingham Nottingham UK; ^3^ Department of Inflammation and Ageing, MRC‐Versus Arthritis Centre for Musculoskeletal Ageing Research University of Birmingham Birmingham UK; ^4^ National Institute for Health and Care Research (NIHR) Birmingham Biomedical Research Centre Birmingham UK

**Keywords:** nerve repair, neuritin 1, plasticity, sensory nerve

## Abstract

Neuritin 1 (NRN1) has emerged as a multifaceted regulator of synaptic plasticity, neuronal excitability and structural remodelling. This review synthesises knowledge of NRN1 function across the central and peripheral nervous systems, with a focus on its roles in sensory neurones and neuronal repair following injury. We discuss evidence that NRN1 interacts with classical neurotrophic pathways, including brain‐derived neurotrophic factor (BDNF) and nerve growth factor (NGF), while engaging distinct cellular mechanisms that span activity‐dependent trafficking, modulation of calcium and potassium channel function and regulated local axonal mRNA translation. Accumulating data indicate that NRN1 contributes to injury‐induced plasticity and functional recovery through both cell‐autonomous neuronal mechanisms and non‐cell‐autonomous signalling involving glial and stromal cells. In long‐projecting sensory axons, regulated transport and local translation of *Nrn1* mRNA position NRN1 as a spatially restricted effector of axonal growth, excitability and regeneration. Dysregulation of NRN1 expression and signalling has been implicated in pathological contexts including neurodegeneration, diabetic peripheral neuropathy and inflammatory pain, where restoration of NRN1 activity promotes axonal integrity, Schwann cell survival and neurotrophic support. Beyond neurons, NRN1 also modulates inflammatory and angiogenic pathways, including VEGF and CXCR4 signalling, linking neuronal plasticity to broader tissue and immune responses. Together, these findings support a model in which NRN1 acts as a molecular integrator of neurotrophic, metabolic and injury‐associated signals, coordinating plasticity while also presenting potential routes to maladaptive sensitisation. We highlight key mechanistic and translational challenges that must be addressed to harness NRN1 biology therapeutically aimed at enhancing neuronal repair while limiting persistent sensory dysfunction.

## Graphical abstract

Neuritin 1 (NRN1) is a multifaceted regulator of synaptic plasticity, neuronal excitability and structural remodelling, acting as a molecular integrator of neurotrophic, metabolic and injury‐associated signals that coordinate adaptive plasticity. Therapeutic targeting of NRN1 biology therefore has the potential to optimise neuronal repair while limiting persistent sensory dysfunction.

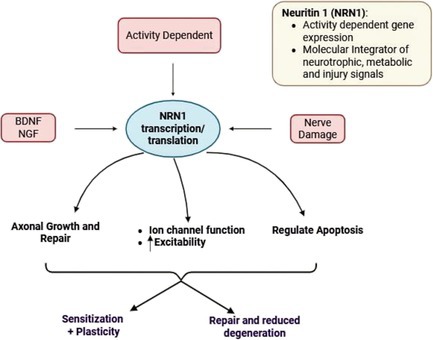

**Abbreviations**
ad
: Alzheimer's disease
Akt: Protein kinase B
AMPA: α‐Amino‐3‐hydroxy‐5‐methyl‐4‐isoxazolepropionic acid
Arc: Activity regulated cytoskeleton‐associated protein
Bcl‐2: B‐cell lymphoma 2
BDNF: Brain‐derived neurotrophic factor
Ca^2+^
: Calcium
cAMP: Cyclic adenosine monophosphate
CaN: Calcineurin
Cav3.3: Low‐voltage‐activated Ca_v_3 channels
CGN: Cerebellar granule neurones
CGP15: Candidate plasticity gene15
CREB: Cyclic AMP responsive element binding protein
CXCL12: C‐X‐C motif chemokine ligand 12
CXCR4: C‐X‐C chemokine receptor type 4
DNA: Deoxyribonucleic acid
DRG: Dorsal root ganglia
ERK: Extracellular signal‐regulated kinase
EP: Ecliptic pHluorin
GAP‐43: Growth‐associated protein 43
GDNF: Glial cell line‐derived neurotrophic factor
GFP: Green fluorescent protein
GPI: Glycosylphosphatidyl inositol
I_A_
: Transient outward potassium current
IGF1: Insulin‐like growth factor 1
IR: Insulin receptor
Kv4.2: Potassium voltage‐gated channel subfamily D member 2
MCAO: Middle cerebral artery occlusion
MEK: Mitogen‐activated protein kinase kinase
MiR‐199a: MicroRNA 199a
mRNA: Messenger ribonucleic acid
MTOR: Mammalian target of rapamycin
NEURL1: Neutralised‐like 1
NF‐200: Neurofilament 200
NFATc4: Nuclear factor of activated T‐cells 4
Nfatc4‐/: Knockout of nuclear factor of activated T‐cells 4
NGF: Nerve growth factor
NMDA: N‐methyl‐D‐aspartate
NRN1: Neuritin1
NT‐3: Neurotrophin3
OA: Osteoarthritis
PARP1: Poly[ADP‐ribose] polymerase 1
PI3K: Phosphoinositide 3‐kinase
PKA: Protein kinase A
PSD95: Postsynaptic density protein 95
p‐Stat3: Phosphorylated signal transducer and activator of transcription‐3
p‐Akt: Phosphorylated protein kinase B
RGC: Retinal ganglion cell
siRNA: Small interfering RNA
STAT3: Signal transducer and activator of transcription 3
SYN‐38: Synaptophysin
TrkB: Tropomyosin receptor kinase B
UTR: Untranslated region
VEGF: Vascular endothelial growth factor
WD: Wallerian degeneration
WT: Wild type

## 1 Introduction

**FIGURE 1:**
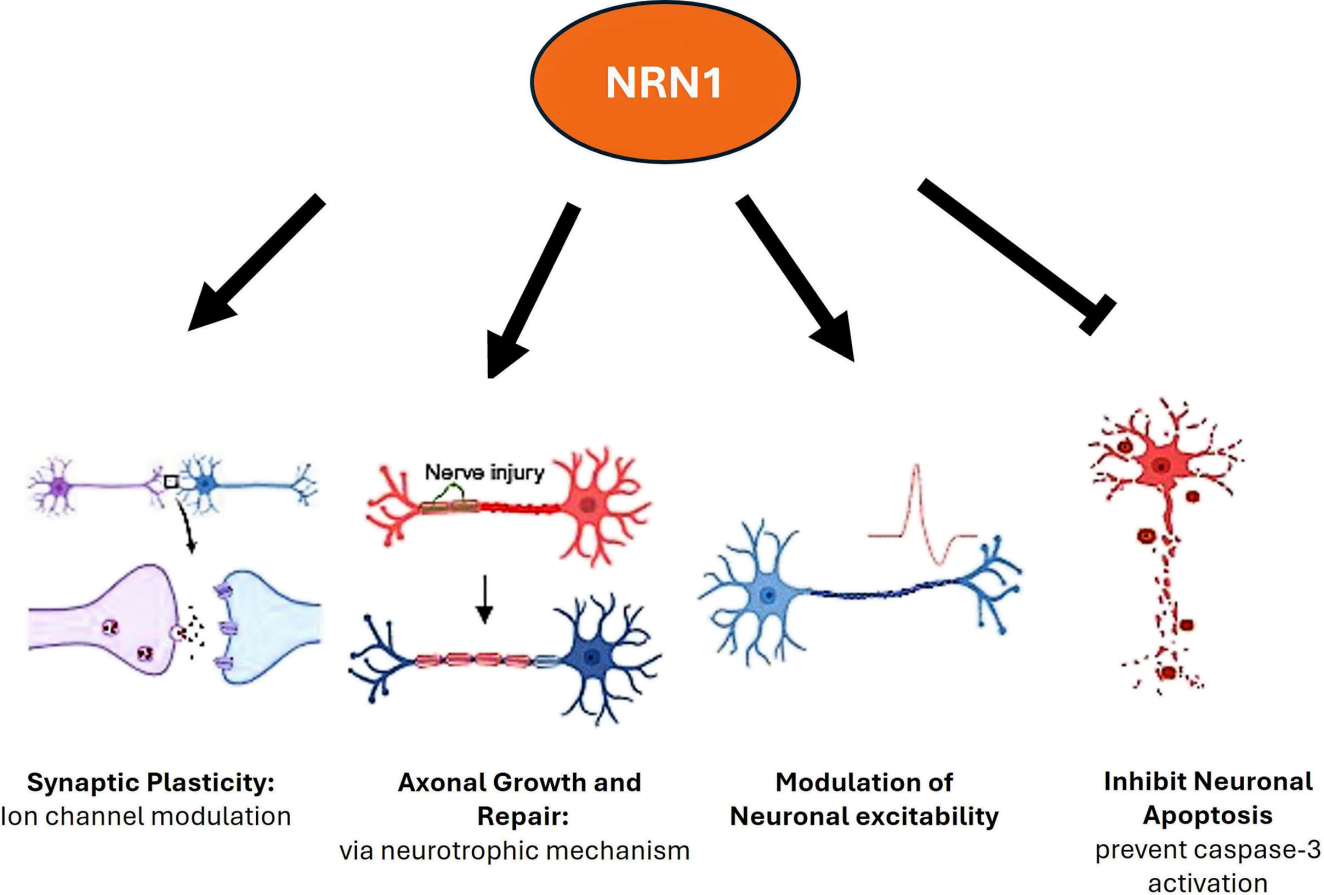
Role of neuritin (NRN1) in synaptic plasticity and sensory nerve function. NRN1‐mediated synaptic plasticity is mediated by multiple ion channels, including NMDA receptors and voltage‐gated calcium channels (Fujino et al. 2003). NRN1 promotes axonal growth and repair. NRN1 has complex effects on excitability via changes in both glutamate signalling and increased potassium currents (Lu et al. 2017; Yao et al. 2012). NRN1 can inhibit neuronal apoptosis by preventing activation of caspase‐3 (Putz et al. 2005; Yao et al. 2012, 2016) (created with Biorender.com).

The development, activity, plasticity and survival of the peripheral and central nervous system, including the axonal remodelling and regeneration of sensory neurons, are regulated by the complex interactions of multiple neurotrophins and associated molecules (Richner et al. 2014; Li et al. 2020). Recently, a role of neuritin 1 (NRN1) in these processes has come to the fore. NRN1, originally known as candidate plasticity gene15 (CPG15), was identified in a screen of plasticity‐related genes in the rat brain (Nedivi et al. 1993). NRN1, encoded by *Nrn1*, was initially described as a glycosylphosphatidylinositol (GPI)‐anchored protein involved in activity‐dependent dendritic growth (Naeve et al. 1997; Cappelletti et al. 2007; Nedivi et al. 2001; Di Giovanni et al. 2005; Nedivi et al. 1998), yet it is increasingly apparent that additional modes of NRN1 localisation and signalling might contribute to its broader biological functions (Figure 1). In cortical primary neurones, NRN1 was identified as an immediate early gene induced by Ca^2+^ influx, requiring activation of NMDA receptors and L‐type Ca^2+^ channels (Fujino et al. 2003). Early studies also suggested that NRN1 may function as a soluble ligand in addition to its membrane‐anchored form (Naeve et al. 1997); however, a canonical receptor responsible for mediating its effects has not yet been identified. Extending these observations beyond somatodendritic compartments, subsequent studies demonstrated that *Nrn1* mRNA is actively transported into axons and locally translated in sensory neurons, particularly following nerve injury, revealing a spatially restricted mode of NRN1 regulation that supports axonal growth and regeneration (Merianda et al. 2013).

Here, we review the literature reporting a role for NRN1 in both peripheral and central nervous system function, with particular emphasis on its involvement in the modulation of excitatory synaptic structures which is fundamental to neural integrity and plasticity. We discuss the role of NRN1 in regulating sensory neuronal excitability via modulation of calcium and potassium currents, and its influence on axonal integrity and repair in models of diabetic peripheral neuropathy. The functional implications of NRN1's role in modulating synaptic circuitry and plasticity and the interplay between NRN1 and widely studied neurotrophic factors, such as nerve growth factor (NGF) and brain‐derived neurotrophic factor (BDNF) (Almodóvar‐Payá et al. 2024; Cappelletti et al. 2007) are highlighted. In this context, we propose that the collective literature supports further investigation of NRN1 as a therapeutic target to normalise sensory nerve excitability, with the potential to alleviate maladaptive sensory nerve signalling (Figure 1).

### 1.1 NRN1: Established Roles in Neurodevelopment

Since the first identification of NRN1 (Nedivi et al. 1993), its role in mediating neuronal plasticity, an essential mechanism supporting the development, maturation and refinement of the nervous system, has been consolidated (Zhou and Zhou 2014, Figure 1). As summarised in previous work (Leslie and Nedivi 2011), NRN1 regulates the structure and development of the visual system, consistent with a role in the formation of activity‐dependent synaptic connections (Corriveau et al. 1999; Lee and Nedivi 2002; Picard et al. 2014). NRN1 now has an established role in the development and maturation of hippocampal neuron connectivity (Lee et al. 2021; Son et al. 2012). For example, in the hippocampal subicular regions, NRN1 promotes neurite growth, which is also regulated by TrkB‐mediated BDNF signalling and neurotrophin 3 (Naeve et al. 1997). More recently, NRN1 knockout was shown to reduce spine stabilisation in the mouse visual cortex, delay axon and spine arbor development, and synapse maturation essential for efficient learning in the hippocampus (Fujino et al. 2011).

The effects of NRN1 on neurite growth have been shown to be mediated by NRN1 acting as an upstream and negative regulator of neutralised‐like 1 (NEURL1), which inhibits Notch signalling to promote neurite growth (Zhang et al. 2017, Figure 2). The stimulatory effects of NRN1 on neurite growth were blocked by NEURL1, indicating the interplay between these molecules in regulating neuronal development and regeneration (Zhang et al. 2017). More recently, NRN1 was shown to inhibit NEURL1 activity by modulating the target protein Jagged1 (ligand for notch1) (Zhu et al. 2023). While inhibition of NEURL1 by NRN1 to regulate the Notch signalling pathway has emerged as a model, the details of this pathway may vary across cell types or developmental stages, warranting further investigation. In addition to these pathways, NRN1 overexpression has been shown to promote retinal ganglion cell survival and axonal regeneration via activated p‐Stat3 and p‐Akt1 signalling transduction pathways (Huang et al. 2021).

**FIGURE 2:**
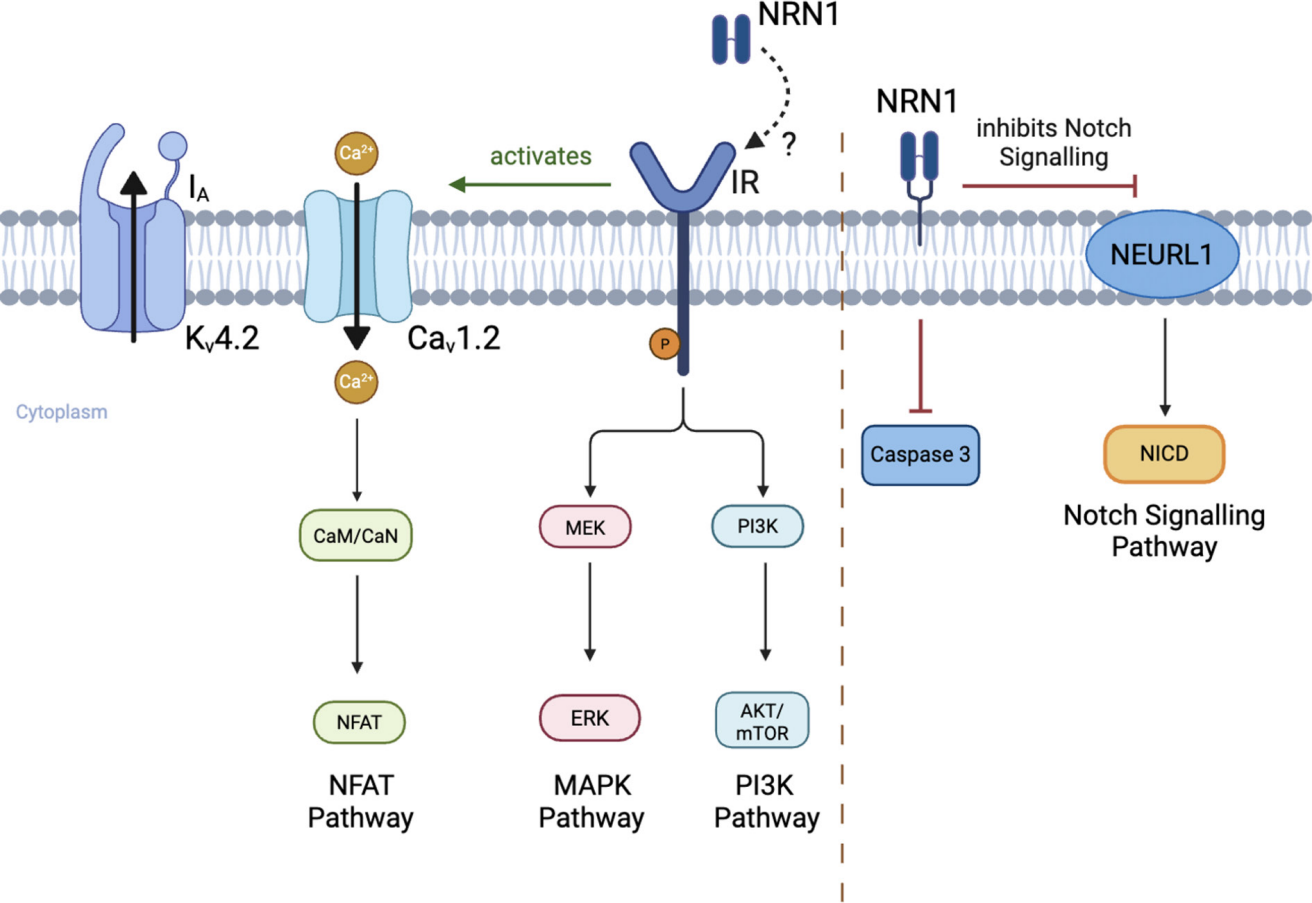
Schematic representation of NRN1‐mediated signalling pathways and their role in neuronal function. (a) NRN1 (Neuritin 1) has been shown to engage multiple intracellular signalling pathways, including NFAT, MAPK/ERK and PI3K/AKT/mTOR cascades, through mechanisms involving calcium‐dependent signalling and receptor‐mediated processes, including functional interactions with the insulin receptor (IR). (b) NRN1 in membrane bound form inhibits caspase‐3 activity, thereby reducing neuronal apoptosis. NRN1 also regulates Notch signalling through NEURL1, which suppresses the formation of the Notch intracellular domain (NICD), influencing neuronal differentiation (Yao et al. 2016; Zhang et al. 2017; Gao et al. 2014).

In *Xenopus* optic tectal neurons, NRN1 (described in this study as CPG15) expression led to the recruitment of functional AMPA receptors to synapses, promoting retinotectal synapse maturation (Cantallops et al. 2000). The expression of a truncated form of NRN1, which lacks the GPI domain, eliminated the promotion of axo‐dendritic growth and synaptic maturation (Nedivi et al. 1998; Cantallops et al. 2000). While these results suggest that the GPI anchor is essential for the membrane‐bound form of NRN1 to regulate neurodevelopment, in early development NRN1/CPG15 also has functional effects in the soluble form, acting as a survival factor for cortical progenitor cells (Putz et al. 2005). Supporting this protective role, endogenous CPG15 can rescue cultured cortical neurons from apoptosis during development (Putz et al. 2005).

### 1.2 NRN1 Modulates Neuronal Excitability via Regulation of Ion Channels

Alongside established roles in development, recent studies suggest that NRN1 can regulate neuronal excitability via interactions with key ion channels that modulate synaptic activity (Figures 1 and 2), predominantly reported for cortical neurones, but also observed in cerebellar granule neurons (CGN). For example, membrane‐anchored NRN1 increased the frequency of miniature excitatory postsynaptic currents and glutamate release in a mouse medial prefrontal cortical preparation, an effect blocked by T‐type Ca2+ channel inhibition (Lu et al. 2017). In this study, NRN1‐driven increases in the surface expression of Cav3.3 were blocked by inhibition of insulin receptors or MEK/ERK activity (Lu et al. 2017). Exogenous application of NRN1 enhanced intracellular calcium by increasing membrane expression of Cav1.2 and Cav1.3 channels in CGN neurons (Yao et al. 2016; Zhao et al. 2018, Figures 1 and 2) and Cav3.3 channels in the mouse medial prefrontal cortex (Lu et al. 2017). NRN1‐induced elevations in intracellular calcium potentiated voltage‐gated IA current densities and Kv4.2 channel expression in CGN neurons (Yao et al. 2012, 2016).

Proteomic analysis has suggested a potential interaction between NRN1 and AMPA receptors (Schwenk et al. 2012, 2014), with functional studies supporting a direct interaction between them (Subramanian et al. 2019). In the latter study, post‐synaptic NRN1 was required cell autonomously for the activity‐dependent recruitment of PSD95 to newly formed dendritic spines, and cell‐autonomous NRN1 expression was shown to act as an experience‐dependent facilitator of PSD95 recruitment and spine stabilisation (Subramanian et al. 2019).

### 1.3 The Role of NRN1 in Neuronal Repair

There is mounting evidence that NRN1 plays a role in the adult brain under pathological conditions, where its re‐expression appears to attempt to recapitulate developmental growth and repair pathways (Figure 1). For example, middle cerebral artery occlusion in rats was associated with increased expression of NRN1, BDNF and Arc mRNA in the peri‐infarct cortex and dentate gyrus, regions that are implicated in post‐stroke plasticity and functional recovery (Rickhag et al. 2007). Similarly, both mRNA and protein levels of NRN1 were upregulated in the cortex and hippocampus following induction of a transient global ischaemia model, also supporting an involvement of NRN1 in injury‐induced neuronal responses (Gao et al. 2014). These findings suggest that NRN1 can be reactivated in the mature brain under conditions of injury or stress, where NRN1 may potentially contribute to synaptic remodelling, circuit reorganisation or neuroprotective mechanisms reminiscent of its developmental functions. This concept is supported by the demonstration that overexpression of NRN1, alongside other hippocampal protein expression of reparative molecules GAP‐43 and SYN‐38 and NF‐200, promoted neuroregenerative capacity and improved recovery of spatial learning and memory in a model of acute ischaemia–reperfusion‐induced brain injury in mice (Wan et al. 2020). In this mouse model, hippocampal overexpression of NRN1 significantly reduced degradation of the DNA repair factor poly[ADP‐ribose] polymerase 1 (PARP1) in the hippocampus (Wan et al. 2020). In addition, overexpression of NRN1 improved sensory motor dysfunction and reduced brain lesions and oedema in an intracerebral haemorrhage (ICH) mouse model (Lu et al. 2021).

Consistent with a neuroadaptive role, NRN1 has also been implicated in maintaining functional connectivity in the human brain. Cortical and hippocampal NRN1 expression was significantly downregulated in the brains of Alzheimer's disease (AD) patients, compared to age‐matched controls (Choi et al. 2014). These changes were considered functionally relevant, as lentiviral mediated expression of NRN1 restored dendritic spine density and synapse maturation in primary hippocampal neuron cultures derived from a transgenic mouse model of AD, returning them to levels observed in wild‐type neurons (Choi et al. 2014). Moreover, lentiviral‐mediated expression of NRN1 ameliorated the learning and memory impairments in the Tg2576 mice, which was suggested to result from improved spine density and synapse maturation (Choi et al. 2014). In line with these findings, hippocampal neuron cultures from Tg2576 mice exhibited decreased levels of NRN1 mRNA and reduced dendritic complexity compared to WT controls, which was reversed by exogenous application of NRN1 peptide (An et al. 2014). Consistent with a post‐transcriptional mechanism contributing to NRN1 loss, related studies have shown that hippocampal miR‐199a expression is increased in a mouse model of AD, and that miR‐199a directly downregulates NRN1 by targeting the Nrn1 3′ untranslated region (UTR) (Song et al. 2020). Mechanistic studies further show that NRN1 counteracts amyloid‐β42‐induced dendritic spine degeneration and neuronal hyperexcitability in primary rat hippocampal neuron cultures, supporting the concept that NRN1 contributes to synaptic resilience and neuronal repair under pathological conditions (Hurst et al. 2023).

### 1.4 Roles of NRN1 in Long‐Projecting Axons: Local Translation and Sensory Neurones

Extending beyond synaptic maintenance and strengthening in complex brain regions such as the hippocampus, NRN1 also plays a role in the response to injury in long‐projecting axons. For example, NRN1 expression in the retina was upregulated following optic nerve injury in mice (Azuchi et al. 2018). Furthermore, NRN1 knockout resulted in more severe retinal ganglion cell loss and reduced activation of Akt and ERK, important mediators of pro‐survival signalling in retinal ganglion cells, suggesting a neuroprotective role of NRN1 in the retina (Azuchi et al. 2018).

Expression of a fluorescently tagged NRN1 fusion protein in *Xenopus* tadpole optic tectal explants and retinal ganglion cells enabled in vivo visualisation of NRN1 trafficking and showed that its delivery to the axonal surface is activity‐ and calcium‐dependent (Cantallops and Cline 2008). Unlike other GPI‐anchored proteins, NRN1 dynamically cycles to and from the axonal membrane via a calcium‐dependent mechanism and maintains an intracellular axonal pool associated with vesicles and endosomes. These properties, together with its predominant axonal localisation and rapid activity‐dependent surface delivery, support a role for NRN1/CPG15 as a trophic signalling molecule under tight spatiotemporal control, coordinating synaptic function with pre‐ and postsynaptic structural plasticity in an activity‐dependent manner (Cantallops and Cline 2008). Collectively, these findings identify axons as a critical compartment for NRN1 signalling, raising the possibility that NRN1 function is tightly coupled to local, activity‐dependent mechanisms that support axonal adaptation. Sensory neurones possess exceptionally long axons that relay complex signals from peripheral targets to the spinal cord and brain, underpinning their essential role in the detection of external somatic sensations, including pain. It is well recognised that local mRNA translation is a central tenet of axon biology and neuronal function, enabling spatially restricted protein synthesis that allows axons to rapidly adapt to environmental cues without requiring input from the soma. These mechanisms are particularly relevant in the peripheral nervous system, where the long distances between neuronal somata and distal axon terminals impose distinct logistical constraints on rapid functional adaptation, for example following injury. Indeed, local translation in sensory neurones is increasingly recognised as a major contributor to injury responses, regenerative growth and peripheral sensitisation and pain modulation (Khoutorsky and Price 2018; Gale et al. 2022).

Notably, NRN1 has a role in sensory nerve development and function, as expression of NRN1‐GFP constructs in dorsal root ganglion (DRG) neurones increased axon growth, whereas depletion of *Nrn1* mRNA significantly reduces growth (Merianda et al. 2013). Following sciatic nerve injury, NRN1 mRNA and protein levels are increased within sciatic nerve axons, with a concomitant decrease in DRG neuronal cell bodies, indicating a pronounced redistribution of NRN1 from somatic to axonal compartments during injury responses (Merianda et al. 2013). Importantly, this shift in localisation was mediated by elements within the *Nrn1* 5′UTR, identifying regulated mRNA transport and local axonal translation as key mechanisms underlying NRN1 function in injured sensory neurons (Merianda et al. 2013). Recombinant NRN1 has been shown to promote hindlimb function and axonal regeneration in an acute model of spinal cord injury, and NRN1 administration during the early stages post‐injury increased NF200 and Gap‐43, markers for neuronal function and axonal transport (Gao et al. 2016).

### 1.5 Role of RNA Binding Proteins in NRN1 Function

Beyond demonstrating axonal localisation, emerging work has begun to define the cis‐ and trans‐acting mechanisms that govern *Nrn1* mRNA trafficking and translation. In sensory neurons, axonal targeting of *Nrn1* is driven predominantly by 5′UTR‐encoded localisation signals, in contrast to hippocampal neurons where 3′UTR elements can be sufficient, suggesting cell‐type‐specific deployment of *Nrn1* localisation ‘zipcodes’ and ribonucleoprotein (RNP) programmes (Merianda et al. 2013). Consistent with this, axonal localisation of *Nrn1* mRNA is sensitive to RNA‐binding protein availability and competition; endogenous *Nrn1* and *Gap43* transcripts compete for binding to the ELAV‐family protein HuD, such that higher levels of *Nrn1* are required to displace *Gap43* 3′UTR interactions, while depletion of *Gap43* enhances *Nrn1* 3′UTR‐dependent axonal localisation (Gomes et al. 2017). Together, these findings place *Nrn1* within a HuD‐regulated post‐transcriptional programme that governs its axonal availability and may influence axonal growth and regeneration. More broadly, affinity proteomic analyses of axonal localisation motifs have identified hnRNP H1, hnRNP F and hnRNP K as injury‐responsive RNA‐binding proteins that associate with *Nrn1* motifs, with axotomy enhancing their axonal transport and depletion impairing axonal mRNA abundance, local protein synthesis and regenerative growth. Together, these findings support the existence of coordinated axonal RNA regulons in which *Nrn1* operates as part of a wider post‐transcriptional programme driving axonal adaptation and regeneration (Lee et al. 2018).

The functional relevance of the redistribution and local synthesis of NRN1 has yet to be fully resolved but is likely to reflect a requirement for spatially restricted NRN1 production to support rapid axonal responses following injury. Locally synthesised NRN1 may enable acute modulation of membrane excitability, cytoskeletal dynamics, and retrograde signalling capacity, thereby influencing both regenerative growth and the thresholds governing peripheral and central sensory neuron excitability. Given the emerging importance of local protein synthesis in injury‐induced plasticity, peripheral sensitisation, and sensory nociceptor function, elucidating the mechanisms that regulate *Nrn1* mRNA trafficking and translation represents an important frontier in understanding how NRN1 contributes to sensory neuron plasticity and persistent pain.

### 1.6 The Contributions of NRN1 to Sensory Neuron Function in Diabetic Peripheral Neuropathy

Beyond acute injury responses, dysregulation of NRN1 has also been implicated in sensory nerve pathology under chronic metabolic stress, most notably in diabetic peripheral neuropathy (DPN). DPN is a common and debilitating complication of diabetes, frequently characterised by pain in the extremities, sensory loss, foot ulceration, and, in severe cases, amputation, collectively leading to a marked reduction in quality of life due to peripheral nervous system dysfunction (Elafros et al. 2022). In this context, alterations in neurotrophic support, axonal integrity and sensory neuron excitability are central features of disease progression, positioning NRN1 as a candidate molecular integrator of metabolic and neurotrophic signalling pathways relevant to both nerve degeneration and maladaptive pain. Reduced axonal transport and expression of NRN1 have been linked to NGF dysfunction in a rat model of diabetes (Karamoysoyli et al. 2008). In vitro, NGF treatment increased the transcription and translation of NRN1 in sensory neurons and siRNA knockdown of NRN1 abolished NGF‐mediated neurite outgrowth (Karamoysoyli et al. 2008). In a separate study, mRNA expression levels of both NRN1 and NGF were significantly reduced in the sciatic nerve from diabetic rats (Ma et al. 2018), which was suggested to contribute to the pathogenesis of diabetic neuropathy.

NRN1 has also been implicated in signalling pathways which may be relevant to sensory nerve excitability in the context of diabetes. NRN1 activates the insulin receptor (IR) and increased the density of transient outward potassium currents (I_A_) via an up‐regulation of the voltage‐gated K^+^ channel Kv4.2 in cerebellar granule neurones (Yao et al. 2012). In this study, NRN1‐mediated activation of IR and mTOR/ERK (mammalian target of rapamycin/extracellular signal‐regulated kinase) signalling pathways increased mRNA and protein expression of Kv4.2 (Figure 2). More recent work demonstrated a requirement of Ca2^+^/calcineurin (CaN)/nuclear factor of activated T‐cells (NFATc4) in the NRN1/IR pathway in CGN and HeLA cells (Yao et al. 2016). NFATc4 was recruited to the Kv4.2 gene promoter and the effects of NRN1 overexpression on neuronal excitability and dendritic spine formation were abrogated in Nfatc4−/− mice (Yao et al. 2016).

The potential roles of NRN1 in diabetes are not limited to neurones as NRN1 is expressed as both a membrane form and a dominant soluble form in Schwann cells. High glucose concentrations linked to diabetes downregulate NRN1 levels, an effect associated with Schwann cell apoptosis (Min et al. 2012; Yan et al. 2018) and potentially negatively impacts sensory nerve function. NRN1 has been shown to accelerate Schwann cell dedifferentiation and be a positive regulator of Wallerian degeneration (Liu et al. 2024). In addition, in this model of nerve injury, NRN1 enhanced the expression of neurotrophic factors such as NGF, GDNF, and BDNF, as well as the phagocytic and secretory activity of Schwann cells, collectively accelerating the subsequent regenerative process. In experimental models of diabetes, NRN1 mRNA and protein levels are lowered, and exogenous application of NRN1 was shown to increase cell viability and decrease apoptosis of Schwann cells by enhancing the Bcl‐2 level and reducing caspase‐3 activity (Yan et al. 2018; Xi et al. 2020). NRN1 interactions between sensory nerves and Schwann cells have been suggested as co‐culture of diabetic DRG neurons with Schwann cells pre‐treated with exogenous NRN1 ameliorated reductions in DRG neurite outgrowth and NGF levels (Xi et al. 2020). Administration of insulin growth factor (IGF‐1), which attenuates glucose‐induced apoptosis of Schwann cells, has been associated with a reduction in NRN1 levels (Yan et al. 2018). Collectively, these findings highlight NRN1 as an integrator of metabolic and neurotrophic signalling in both neurons and Schwann cells, with emerging relevance to the pathogenesis of diabetic peripheral neuropathy and more broadly regenerative and plasticity processes in other neural systems.

### 1.7 NRN1 Regulation of Inflammatory Signalling and Osteoarthritis Pain

NRN1 is emerging as a modulator of inflammatory signalling pathways that contribute to sensory nerve sensitisation and pain, particularly in the context of osteoarthritis (OA). A key pathway implicated in neuropathic pain and central sensitisation involves the chemokine receptor CXCR4 and its ligand CXCL12, which promote inflammatory signalling in the spinal dorsal horn following injury (Liu et al. 2019; Yu et al. 2017). Clinical evidence suggests an association between levels of CXCR4 in synovial fluid and OA patient outcomes (Trajerova et al. 2022), and CXCR4 contributes to osteoarthritis pathology in an experimental model (Wei et al. 2012). The nrn1 gene is co‐expressed with CXCR4 in renal cell carcinoma cells, and NRN1 overexpression significantly increased CXCR4 mRNA levels, although the precise mechanism remains unclear (Kamada et al. 2021). Mechanistic findings indicate that NRN1 functional interacts with the insulin receptor, activating the transcription factor NFATc4, which is known to bind to the CXCR4 promoter and upregulate expression (Yao et al. 2016; Cole et al. 2020). Thus, NRN1‐dependent regulation of CXCR4, a critical mediator of pain signalling, warrants further investigation to clarify its potential role in sensory nerve sensitisation.

Vascular endothelial growth factor (VEGF) is well established as promoting angiogenesis and also playing important roles in osteoarthritis and associated pain (Hamilton et al. 2016), inflammatory processing, and sensitisation of sensory neurones (Llorián‐Salvador and González‐Rodríguez 2018; Zhang et al. 2019). NRN1 overexpression in pulmonary endothelial cells increases VEGF receptor (VEGFR) mRNA and protein levels, while also inhibiting Notch‐associated factors involved in vascular and inflammatory signalling (Zhang et al. 2019; Yang et al. 2021). Additionally, NRN1 modulates melanoma signalling via Notch and STAT3 pathways, leading to an upregulation of downstream targets such as VegfA (Devitt et al. 2024). Although these pathways have not yet been fully characterised in the context of sensory nerve sensitisation or OA pain, they suggest a broader role for NRN1 in coordinating transcriptional networks that influence inflammation and neuronal responsiveness.

Osteoarthritis is characterised by chronic joint pain originating from the diseased tissue. NRN1 mRNA expression was shown to be elevated in the synovium at sites of patient‐reported pain in early‐stage OA (Nanus et al. 2021). Moreover, the secretome from fibroblast‐like synoviocytes isolated from these painful synovial tissues promoted neurite outgrowth and neuronal survival in rat primary DRG cultures (Nanus et al. 2021). These findings suggest that synovial NRN1 may act as a peripheral integrator of sensory nerve sensitisation through paracrine interactions between synoviocytes and sensory neurons during OA‐associated inflammation. Further studies are required to directly link NRN1‐mediated effects to pain behaviour in models of pathology, such as OA pain. Nevertheless, these data highlight NRN1 as a promising target for future investigations aimed at understanding and potentially mitigating inflammatory and arthritic pain.

## 2 Conclusion

Collectively, the literature reviewed here positions NRN1 (also known as CPG15) as a multifunctional regulator of neuronal plasticity that operates across developmental, injury‐induced and disease‐associated contexts (Figure 1). Originally defined by its role in activity‐dependent synaptic maturation, NRN1 is now recognised to influence neuronal excitability, axonal growth and repair through a diverse array of mechanisms, including modulation of ion channel function, activity‐dependent trafficking and local axonal mRNA translation. Importantly, these processes engage both cell‐autonomous mechanisms within neurons, such as local translation and intrinsic excitability control, and non–cell‐autonomous pathways, including cell–cell signalling. This mechanistic diversity places NRN1 at the intersection of neurotrophic, metabolic and activity‐dependent signalling networks that shape sensory neuron adaptation, sensitisation, and recovery following injury. Dysregulation of NRN1 in pathological states such as neurodegeneration, diabetic peripheral neuropathy and inflammatory pain further supports a role for NRN1 as a determinant of neuronal vulnerability versus resilience.

From a translational perspective, NRN1 presents both significant opportunities and clear challenges as a therapeutic target. Its capacity to promote axonal regeneration, preserve synaptic integrity, and modulate neuronal excitability highlights its potential for restoring neural function; however, these effects are mediated through context‐dependent cellular mechanisms that vary across neuronal and non‐neuronal cell types. The coexistence of membrane‐bound and soluble NRN1, the engagement of distinct downstream signalling pathways and the contribution of both cell‐autonomous and paracrine actions raise important questions about how NRN1 activity might be selectively harnessed in vivo. In particular, the dissociation of reparative and homeostatic NRN1 signalling from pathways that may contribute to maladaptive plasticity or persistent pain will be essential for therapeutic development.

Key outstanding challenges therefore include defining the receptors and downstream effectors that mediate NRN1 function in specific cellular contexts, resolving how NRN1‐dependent RNA regulatory programmes are engaged following injury or metabolic stress, and determining how neuron‐intrinsic and non‐cell‐autonomous NRN1 signalling converge at the level of neuronal circuits. At the same time, emerging insights into NRN1‐regulated axonal translation, cell–cell interactions, and inflammatory signalling provide concrete opportunities for therapeutic innovation, including strategies that target defined NRN1‐associated signalling nodes or cellular compartments rather than global NRN1 activation. Addressing these challenges will be critical for translating NRN1 biology into rational, context‐specific interventions aimed at enhancing neuronal repair while limiting maladaptive neuronal plasticity.

## Author Contributions

**Jyoti Agrawal:** conceptualisation; formal analysis; writing – original draft; writing – review and editing. **Mar Vives Escola:** conceptualisation; formal analysis; writing – original draft; writing – review and editing. **Simon W. Jones:** conceptualisation; formal analysis; writing – original draft; writing – review and editing. **Victoria Chapman:** conceptualisation; formal analysis; writing – original draft; writing – review and editing. **Federico Dajas‐Bailador:** conceptualisation; formal analysis; writing – original draft; writing – review and editing.

## Funding

This work was supported by Versus Arthritis, 20777, 23292, and the Medical Research Council, MR/W026961/1.

## Conflicts of Interest

The authors declare no conflicts of interest.

## Data Availability

This review does not have any data associated with it.
